# US Hospital Characteristics Associated With Price Transparency Regulation Compliance

**DOI:** 10.1001/jamahealthforum.2022.1702

**Published:** 2022-06-24

**Authors:** Yunan Ji, Edward Kong

**Affiliations:** 1 Graduate School of Arts and Sciences, Harvard University, Cambridge, Massachusetts; 2 Department of Economics, Harvard University, Cambridge, Massachusetts; 3 Harvard Medical School, Boston, Massachusetts

## Abstract

This cross-sectional study examines associations between characteristics of US hospitals and their compliance with Centers for Medicare & Medicaid Services regulations for transparency of insurance-negotiated prices.

## Introduction

As of January 1, 2021, the Centers for Medicare & Medicaid Services (CMS) required all US acute care hospitals to release the prices they negotiate with insurance plans to make price comparison across hospitals easier for consumers.^1^ We report data on compliance with this requirement for all 4484 acute care hospitals in the US as of December 2021 and explore the association between hospital characteristics and compliance.

## Methods

In this cross-sectional study, we included all nonexempt acute care hospitals in the 2017 American Hospital Association (AHA) survey. We classified a hospital as compliant if it publicly posted any private payer–specific negotiated prices at the service code level. We manually collected compliance data from hospital websites and verified whether payer-specific prices were reported. We concluded our first round of data collection in July 2021 and rechecked all noncompliant hospitals between December 5 and 27, 2021, establishing a final compliance rate for 2021. We obtained hospital-level characteristics from the AHA, the CMS Provider of Services File, and the Dartmouth Atlas of Health Care. The Harvard University institutional review board determined this study was not human participants research, exempted it from approval, and waived informed consent. We followed the STROBE reporting guideline.

Our unit of observation was a hospital system, defined as 1 or more hospitals in the same integrated health system and hospital referral region (HRR).^2^ For each HRR, we used the share of inpatient beds in each hospital system to construct the Herfindahl-Hirschman Index (HHI). Markets with an HHI of 2500 or greater are considered highly concentrated (ie, less competitive). We used bivariate linear regressions to estimate associations between compliance and hospital system characteristics and report results from multivariate regressions. We conducted statistical tests using robust SEs, with significance defined as 2-sided *P* < .05. Data were analyzed using Stata, version 17 (StataCorp LLC).

## Results

The final sample included 2892 hospital systems representing 4484 hospitals in 306 HRRs. The Table shows descriptive statistics at the hospital system level for 2892 hospital systems; mean (SD) compliance was 68% (46%). We found a negative association between compliance and market competitiveness; compliance was higher in less competitive HRRs (scaled effect size, 0.07; 95% CI, 0.03-0.10) and for hospital systems with greater market shares (scaled effect size, 0.08; 95% CI, 0.05-0.11) (Figure). Both associations remained significant when controlling for number of beds. Multihospital systems (effect size, 0.13; 95% CI, 0.09-0.16), for-profit hospitals (effect size, 0.05; 95% CI, 0.01-0.10), and teaching hospitals (effect size, 0.11; 95% CI, 0.03-0.20) had higher compliance. Government hospitals had lower compliance (effect size, −0.06; 95% CI, −0.10 to −0.02), but the association did not remain after controlling for integration into multihospital systems. Hospital systems with more beds had higher compliance (scaled effect size, 0.04; 95% CI, 0.02-0.07), whereas critical access hospitals (effect size, −0.03; 95% CI, −0.07 to 0.01) and those lacking intensive care units (effect size, −0.13; 95% CI, −0.16 to −0.09) had lower compliance.

**Table.** Characteristics of 2892 Hospital Systems

Characteristic	Mean (SD)	Median (IQR)
Compliance^a^^,^^b^	0.68 (0.46)	1 (0-1)
Market competition		
Market concentration, HHI	2328 (1348)	1955 (1416-3016)
Market share, percentage points	10.6 (15.4)	3.8 (1.2-12.7)
Ownership^b^		
Multihospital system	0.44 (0.50)	0 (0-1)
Private		
For-profit	0.18 (0.37)	0 (0-0)
Nonprofit	0.54 (0.49)	1 (0-1)
Government	0.28 (0.44)	0 (0-1)
Size and resources		
Beds, No.	254 (390)	101 (25-302)
Teaching hospital^b^	0.04 (0.18)	0 (0-0)
Critical access hospital^b^	0.33 (0.45)	0 (0-1)
No ICU^b^	0.33 (0.45)	0 (0-1)

Abbreviations: HHI, Herfindahl-Hirschman Index; ICU, intensive care unit.

^a^ For systems in which some hospitals were compliant and others were noncompliant, the mean compliance rate was assigned. This only applied to 72 (2.5%) of all 2892 systems.

^b^ Variables take values of 0 or 1. The mean value represents the proportion of hospital systems in the category among all systems observed.

**Figure.**
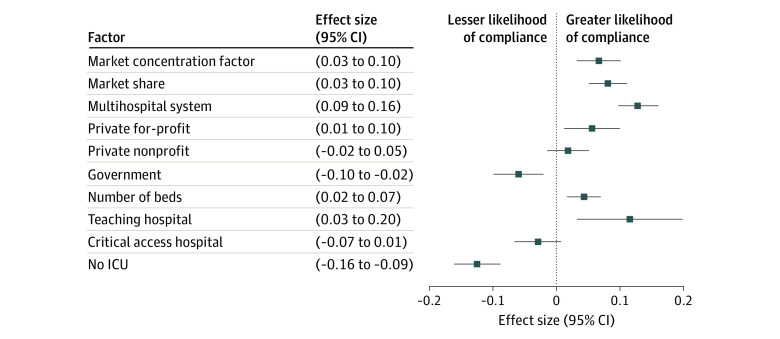
Hospital Factors Associated With Compliance Coefficient magnitudes for market concentration (Herfindahl-Hirschman Index), market share, and number of beds are reported as a 10th- to 90th-percentile increase (Herfindahl-Hirschman Index, 3021; market share, 31 percentage points; and beds, 655). The 95% CIs were computed using robust SEs. Markers indicate effect sizes, with horizontal lines indicating 95% CIs. ICU, intensive care unit.

## Discussion

As of December 2021, 68% of hospitals had released payer-specific negotiated prices, more than 50% higher than rates reported earlier in the year.^3,4,5^ The higher compliance rate may be attributable to reporting delays from technical difficulties or pressure from the media and CMS.

The findings suggest that competition and hospital resources may have a role in determining compliance. Hospitals in the least competitive markets and those with greater market shares had higher compliance rates, consistent with safeguarding of negotiated prices in the presence of greater competition. A prior study^5^ found similar results as of June 1, 2021. In our study, the associations were found through the end of 2021, despite a significant increase in overall compliance.

Our results highlight factors associated with hospital compliance with price transparency regulation. A limitation is that we defined compliance based on availability of payer-specific negotiated prices and omitted other CMS requirements such as availability of a shoppable service tool. A more stringent definition would likely lead to lower measured compliance rates.
